# Supplementary material for: MetaRibo-Seq measures translation in microbiomes
Source: Nat Commun. 2020 Jun 29;11:3268. doi: 10.1038/s41467-020-17081-z (PMC7324362; doi:10.1038/s41467-020-17081-z)
Supplement: Supplementary file 10 — Supplementary Data 7 [file 41467_2020_17081_MOESM10_ESM.zip › File2/Confidence_High_Taxonomy/179508_out.krona.html]

Javascript must be enabled to view this page.

members
magnitude
magnitudeUnassigned
count
unassigned
taxon
rank

179508\_out

10

2
10
superkingdom

1239
10
phylum

186801
10
class

10
186802
order

541000
10
family

8
genus
1263
10

SRS013476\_contig\_number\_contig-100\_24913.129307SRS017433\_contig\_number\_contig-100\_19712.19712SRS017916\_contig\_number\_contig-100\_756.121644SRS043768\_contig\_number\_1845SRS049712\_contig\_number\_contig-100\_1821.136400SRS103987\_contig\_number\_4741SRS104912\_contig\_number\_contig-100\_21065.68741SRS144297\_contig\_number\_contig-100\_7282.84433


SRS077454\_contig\_number\_2462
species
1
2293198

2302958
1
species

SRS148721\_contig\_number\_33210
